# Epstein–Barr Virus Prevalence at Diagnosis and Seroconversion during Follow-Up in Pediatric Inflammatory Bowel Disease

**DOI:** 10.3390/jcm10215187

**Published:** 2021-11-06

**Authors:** Jennifer Bachmann, Giang Le Thi, Annecarin Brückner, Anna-Lena Kalteis, Tobias Schwerd, Sibylle Koletzko, Eberhard Lurz

**Affiliations:** 1 Department of Pediatrics, Division of Gastroenterology and Hepatology, Dr. von Hauner Children’s Hospital, University Hospital, LMU Munich, 80337 Munich, Germany; jennifer.bachmann@med.uni-muenchen.de (J.B.); Thu_Giang.Le_Thi@med.uni-muenchen.de (G.L.T.); annecarin.brueckner@med.uni-muenchen.de (A.B.); tobias.schwerd@med.uni-muenchen.de (T.S.); sibylle.koletzko@med.uni-muenchen.de (S.K.); 2 Max von Pettenkofer Institute & Gene Center, Virology, National Reference Center for Retroviruses, LMU Munich, 80336 Munich, Germany; kalteis@mvp.lmu.de; 3 German Center for Infection Research (DZIF), Partner Site Munich, 38124 Braunschweig, Germany; 4 Department of Pediatric Gastroenterology and Nutrition, School of Medicine, Collegium Medicum University of Warmia and Mazury, 10-561 Olsztyn, Poland

**Keywords:** inflammatory bowel disease, pediatrics, thiopurines, hemophagocytic lymphohistiocytosis, malignancy

## Abstract

Primary Epstein–Barr virus infection in pediatric patients with inflammatory bowel disease during immunomodulation with thiopurines has been associated with increased risk for malignancies or hemophagocytic lymphohistiocytosis. We determined Epstein–Barr virus (EBV) seroprevalence at inflammatory bowel disease (IBD) diagnosis and seroconversion during follow-up in a large single center cohort of children with IBD. EBV serology results and patient characteristics were retrospectively retrieved from the hospital documentation system. EBV seronegative patients at IBD diagnosis were prospectively retested. We report on IBD patients with symptomatic active EBV infection and a complicated disease course, and those diagnosed with malignancy with respect to EBV status and drug exposure. Of 402 patients, 194 (48%) had available EBV serology results at time of IBD diagnosis at a median of 12 years (IQR 9–14 years). Thereof, 102 (53%) were EBV-positive. Of 92 EBV-negative patients, 66 were retested and 17% showed a seroconversion at a mean follow-up time of 4.3 years (SD 3 years). Three children treated with azathioprine experienced acute clinically relevant EBV infection 2, 2.5, and 4 years after IBD diagnosis, two developed signs of hemophagocytic lymphohistiocytosis. Three cases of malignancy occurred in the cohort, though none seemed to be triggered by EBV. In conclusion, almost 50% of pediatric IBD patients were EBV-naïve following diagnosis and may be at increased risk to develop severe EBV infection during immunosuppressive therapy, potentially associated with complications such as hemophagocytic lymphohistiocytosis or malignancy.

## 1. Introduction

Over recent decades, inflammatory bowel disease (IBD) has become a global disease with increasing incidence, particularly in the pediatric population [1,2]. Since there is no cure for IBD, a high proportion of the patients may require immunosuppressive therapies for decades or even lifelong to control the intestinal inflammation. For some of the immunosuppressive drugs, in particular for thiopurines, an increased risk for malignancies has been reported; for some of the newer biologics, the risk is still unknown [3,4,5,6,7,8,9]. Two meta-analyses described a four-to-six-fold higher incidence of lymphoproliferative diseases in IBD patients treated with azathioprine or 6-mercaptopurine compared to patients not receiving thiopurines and the general population [3,4]. The CESAME study, i.e., a French nationwide cohort study including 19,486 IBD patients with a median follow-up of 35 months (IQR 29–40), revealed a significant association between thiopurine therapy and lymphoproliferative disorders with an adjusted hazard ratio of 5.28 (*p* = 0.0007) [5]. In Epstein–Barr virus (EBV) seronegative male IBD patients younger than 35 years of age, the risk to develop early post mononucleosis lymphomas increased from 0.1 to 3 per 1000 patient years [6]. In this patient group, there is also a small but real chance of developing a mostly fatal hepatosplenic T-cell lymphoma, associated with EBV infection [3]. In a prospectively followed cohort of 5766 children with IBD, Hyams et al. reported five cases of hemophagocytic lymphohistiocytosis (HLH), all of whom were children were receiving thiopurines, and four of whom were associated with a primary EBV infection [7]. Biank et al. described an estimated 100-fold greater risk for developing HLH in pediatric IBD patients compared to the local general incidence rate, common characteristics being thiopurine exposure and primary EBV infection [8].

EBV is a human herpesvirus that has been linked to numerous malignancies including endemic (African) Burkitt’s lymphoma, post-transplantation lymphoproliferative disease, Hodgkin’s and B-cell lymphomas, and nasopharyngeal and gastric carcinoma [10,11]. The prevalence of EBV positivity is over 90% in the adult population [10]. In developed countries, only 20% to 25% of children will have acquired EBV by the age of 5 years [12] whereas almost all children are seropositive at 6 years of age in developing countries [10]. Among children and adolescents, a significant decline in age-adjusted prevalence of EBV over the last decades was found [13], and later age at EBV primary infection was associated with higher risk of infectious mononucleosis and severe features [14]. After primary infection, EBV establishes a latent infection in resting memory B-cells, which is characterized by the limited expression of a subset of virus latent genes [15]. Newly infected naïve B cells are controlled by cytotoxic T-lymphocytes and natural killer cells [16]. Thiopurines are cytotoxic for natural killer cells and cytotoxic T-cells and may hamper this control mechanism, and may thereby explain the increased risk for developing certain lymphoma [5].

It is relevant to understand the dynamics of EBV infection in children with IBD in different populations and how this translates into clinical practice. As the risk for complications seems higher in children with primary EBV-infection during therapy with thiopurines, we hypothesize that EBV-naïve children at IBD diagnosis are at higher risk to develop clinically relevant EBV infection or associated morbidities. The aim of this study is to determine the seroprevalence of EBV at the time of IBD diagnosis, the consecutive seroconversion, and EBV-related complications (including malignancies during follow-up at a German tertiary pediatric IBD center).

## 2. Materials and Methods

The cohort consisted of children and adolescents diagnosed with Crohn’s Disease (CD), ulcerative colitis (UC), or IBD-unclassified (IBD-U) before the age of 18 years at our institution between 2003 and 2019. Medical charts were reviewed to identify patient characteristics, EBV status at diagnosis and during follow-up, and received therapies. Special focus was paid to patients with primary EBV infection who showed signs of HLH or on those developing any malignancy during the time period. Detailed case reports are also given. Since 2011, immunological testing was performed as a part of the diagnostic work up in newly diagnosed pediatric patients in our institution. This includes immunoglobulin concentrations (IgA, IgG, IgM), antibody response to common vaccines (diphtheria, tetanus, pneumococcus, H. influenza Typ b), lymphocyte subsets (including natural killer cells), and a dihydrorhodamine flow cytometric test to exclude chronic granulomatous disease (CGD). In cases of very early onset (VEO)-IBD or atypical clinical course, such as HLH, whole exome sequencing was performed within research projects to detect known and unknown monogenetic disorders mimicking IBD. EBV-naïve subjects at IBD diagnosis and during follow-up were prospectively invited for EBV serology testing. Written informed consents of patients and/or caregivers were obtained. The retrospective and the prospective parts of the study were approved by the Ethics Committee of the Ludwig Maximilian University Munich (No. 18-603).

EBV serology testing was comprised of testing for anti-viral capsid antigen (VCA)-IgM, anti-VCA-IgG, anti-Epstein–Barr nuclear antigen (EBNA)-IgG, and anti-Epstein–Barr early antigen (EA)-IgG. EBV serology was tested in blood samples based on enzyme-linked immunosorbent assay (ELISA) technique (2003–2006 DiaSorin ELISA (anti-VCA-IgM, -IgG, anti-EA-IgG, anti-EBNA-IgG), 2004–2018 NovaLisa, Novatec (anti-VCA-IgM), 2006–2018 virion/serion ELISA (anti-VCA-IgG, anti-EA-IgG, and anti-EBNA-IgG), since 2018 DiaSorin LIAISON (anti-VCA-IgM, Anti-VCA–IgG, anti-EA-IgG, and anti-EBNA-IgG). Pipetting was performed by Tecan EvoLyzer (Tecan Group Ltd., Seestrasse 103, 8708 Männedorf, Switzerland) and processing by Siemens BEP III (Siemens Healthcare Diagnostics Products GmbH (formerly Dade Behring), Emil-von-Behring-Str. 76, 35041 Marburg, Germany) since 2004. It was counted as a positive serology if anti-VCA-IgG or anti-VCA-IgM were positive, whereby constellations, including a positive anti-VCA-IgM (without a positive anti-EBNA-IgG), were suggestive of an acute or shortly passed primary EBV infection.

In certain cases, when serological testing was suggestive of primary EBV infection, an in-house quantitative real time polymerase chain reaction (PCR) from blood samples was performed to detect and determine the viral load.

All virological testing (serological and PCR) was carried out in an accredited clinical diagnostic laboratory.

Descriptive statistics with frequency, percentage, and a 95% confidence interval (95% CI) were reported for all patients in the cohort stratified in EBV status. The distribution of EBV status was compared in different strata of nominal variables by using the Chi-square test. Fisher’s exact test was performed where applicable. A univariate logistic regression analysis was performed to determine the association of a considered factor with EBV status. Estimated risks (odds ratio, OR) and 95% CI were reported. *p* values of ≤0.05 were considered statistically significant.

## 3. Results

In total, 402 patients fulfilled the inclusion criteria (Supplemental Figure S1), thereof 207 (51%) patients had undergone EBV testing at any time point (Supplemental Table S1). Compared to the group of patients without available EBV status, no significant differences in gender, diagnosis, and age at diagnosis were found (Supplemental Table S2). However, a difference was seen in the timepoint of diagnosis, with patients without EBV testing being diagnosed with IBD mainly before 2014 (92% vs. 48%).

### 3.1. EBV Status at the Time of IBD Diagnosis

At IBD diagnosis, EBV serology was performed in 194 patients (48%), with a positive EBV serology in 102 (53%) of them (Table 1). All 102 children showed positive anti-VCA-IgG with negative anti-VCA-IgM and anti-EA-IgG, suggestive of a passed infection. Neither type of IBD diagnosis (*p* = 0.963) nor gender (*p* = 0.735) were associated with EBV status. EBV seronegative children were significantly younger (median 11.5 years; IQR 8.0–14.0 years) than EBV-positive ones (median 13 years; IQR 10.0–15.0 years, *p* = 0.005, Supplemental Figure S2). Patients older than 13 years of age had a 2.5 times higher risk of being EBV-positive when compared to patients younger than 9 years old (95% CI 1.21–5.07, *p* = 0.013, Table 2).

### 3.2. EBV Seroconversion during Follow-Up

Of the 92 children with documented negative EBV serology at diagnosis, 66 (72%) were retested after a mean follow-up time of 4.3 (SD 3.0) years and 11 of 66 (17%) patients had seroconverted (Supplemental Table S3). Seroconversion was not related to age, gender, or the type of IBD diagnosis (Table 3). The seroconversion was not significantly related to certain drug exposure, except for a trend in patients with thiopurine therapy compared to no thiopurines (OR 3.71, 95% CI 0.89–15.52, *p* = 0.073).

### 3.3. Case Reports of Children with Primary Acute Symptomatic EBV Infection

Of the 11 children with seroconversion, 3 showed an acute symptomatic EBV infection. A 7-year-old boy with IBD-U acutely presented with fever, reduced general condition, cervical lymphadenopathies, and hepatosplenomegaly, while being treated with azathioprine and 5-ASA for four years. Laboratory tests showed pancytopenia, elevated liver enzymes, increased ferritin (1254 ng/mL, normal range 24–130 ng/mL), and high soluble interleukin-2-receptor levels (6585 kU/L, normal 158–623 kU/L). HLH was considered as five out of eight criteria [17] were met: fever, pancytopenia, splenomegaly, high levels of ferritin, and high soluble interleukin-2 receptor levels. X-linked inhibitor of apoptosis protein (XIAP) deficiency was ruled out by genetic testing. Azathioprine treatment was paused, and laboratory findings normalized within three weeks under close monitoring. Acute EBV infection was confirmed with positive anti-VCA-IgM, anti-EA-IgG, and EBV PCR. One month after full recovery, azathioprine treatment was re-started, but as neutropenia (0.15 G/L) reoccurred, it had to be discontinued. The patient remained in IBD remission with 5-ASA monotherapy for one year when he presented with an acute flare of his colitis, which was successfully treated with infliximab.

A 17-year-old girl presented six years after her CD diagnosis with acute onset of weakness and headaches while being treated with combotherapy with azathioprine for 2.5 years and infliximab for 1.5 years. Laboratory tests showed leucopenia, thrombocytopenia, elevated liver enzymes, and mildly elevated ferritin (554 ng/mL). Azathioprine dose was lowered from 100 mg to 50 mg (1 mg/kg) per day. Anti-VCA-IgG and anti-VCA-IgM were positive but EBV PCR came back negative. The laboratory findings normalized within four weeks and the azathioprine dose was increased to 100 mg per day without further complications. The patient remained in clinical and endoscopic remission until she was transferred to adult care at the age of 21 years.

The third patient with acute clinical EBV infection and later diagnosis of XIAP deficiency has been reported previously [18,19]. In brief, the boy presented at the age of 8 years with recurrent perianal abscesses and bloody diarrhea and was diagnosed with Crohn’s colitis. At that time, EBV serology was negative. His disease course was refractory to azathioprine, anti-TNFα, and cyclosporin A. Two years and eight weeks after stopping azathioprine, but continuing infliximab therapy, he presented with fever, lymphadenopathies, splenomegaly, anemia, leucopenia, and ferritinemia (4361 µg/L). EBV serology revealed positive anti-VCA-IgG (31 U/mL), anti-EA-IgG (32 U/mL), and borderline anti-EBNA-IgG, while anti-VCA-IgM and EBV PCR were negative. Soluble interleukin-2-receptor concentration was not determined. Symptoms and abnormal laboratory findings suggestive of EBV infection resolved spontaneously. Eight months later, follow-up EBV serology revealed only positive anti-VCA-IgG (17 U/mL), but anti-EA-IgG and anti-EBNA-IgG returned negative. One year after the acute EBV infection, the diagnosis of X-linked lymphoproliferative disease type 2 was established. Hematopoietic stem cell transplantation (HSCT) was performed from a human leucocyte antigen (HLA) identical donor. Thereafter, the patient remained off medication and without any symptoms of IBD including a normal colonoscopy.

### 3.4. Case Reports of Children Diagnosed with Malignancy

During the 16-year period, three patients of the cohort were diagnosed with malignancy. A 17-year-old female patient presented with weight loss, night sweats, a cough, and fatigue three years after diagnosis of extensive CD. A CT scan revealed a stage four intrapulmonary lymphoma. At that time, she was treated with combotherapy with azathioprine and infliximab for two years. EBV serology was negative at IBD and at Hodgkin’s lymphoma diagnosis. She was treated by oncology, presented a relapse one year later, but stayed in remission since then.

An 18-year-old male patient diagnosed with CD at the age of 6 years, developed a testicular non-seminomatous germ cell tumor six months after referral to adult care. At the age of 13 years, routine EBV serology revealed positive anti-EA-IgG serology without anti-VCA-IgM or anti-VCA-IgG, while EBV PCR was negative. Follow-up serology four years later showed neither anti-EA-IgG or anti-VCA-IgG positivity; however, at the time of referral to adult care, anti-VCA-IgG was positive for the first time. He was in clinical remission, was treated with infliximab for the last six years, and was thiopurine naïve. Anti-TNFα was paused, and orchiectomy and adjuvant chemotherapy were performed.

The third patient with malignancy was diagnosed with UC and primary sclerosing cholangitis (PSC) at 12 years of age. Annual routine colonoscopy disclosed an adenocarcinoma in the colon by 19 years of age; EBV serology was negative one year earlier. Over the disease course, she was treated with azathioprine, 5-ASA, ursodeoxycholic acid, as well as some courses of prednisolone and adalimumab. This commenced at the age of 15 years, but switched to vedolizumab because of the lack of response 2.5 years later. She was then transferred to the adult program for colectomy.

## 4. Discussion

In this cohort of 402 pediatric patients with IBD, 48% of children were tested for EBV infection at the time of diagnosis and a further 3% at any timepoint during follow-up. Of those with available results at IBD diagnosis, serology indicated passed infection in 53% with older age being the only associated risk factor. Of initially EBV-naïve patients, 17% showed seroconversion when tested as part of this study at a mean follow-up time of 4.3 years. The only drug associated with seroconversion was azathioprine with a 3.7 times higher risk in exposed to non-exposed patients.

Our results confirm previous studies from New York with a low EBV seropositivity in early teenagers with IBD [12] while young adults entering university in Scotland are reported to have high seropositivity of 75% [20]. High infection rates in infants have been reported from Kenya [21] and in native Greenland Eskimo children [22]. Ethnicity and socioeconomic factors, such as poor hygiene and crowding, are associated with early age at EBV infection, while in most Western countries and Japan, an overall decline in seroprevalence of EBV in young children was observed over time [13,23].

Primary EBV infection before 10 years of age is usually asymptomatic or with symptoms of a common cold. In adolescents, the primary infection is more often symptomatic and known as infectious mononucleosis [11]. In our cohort 3 of 11 patients, seroconverting during follow-up showed clinical signs of acute EBV infection severe enough to indicate serologic investigation, while the remaining had either none or milder symptoms not warranting laboratory work up. The three symptomatic patients were on thiopurines and had classic findings, such as myelosuppression, elevated liver enzymes, high ferritin levels, organomegaly, and fatigue. Patients treated with thiopurines, especially those with CD [8], had a 100-fold greater risk for developing HLH, and a high proportion of HLH-cases were associated with acute EBV infection [7]. Therefore, we suggest that, following IBD diagnosis, EBV status should be assessed, and when HLH-suggestive findings occur, acute EBV infection or reactivation must be excluded, which is supported by an ECCO statement from 2014 [24]. Others question this pre-emptive serologic testing approach, as no cost-effectiveness analysis was performed to support this strategy compared to the post solid organ transplant situation. In adults, incident rates of HLH and early post mononucleosis lymphomas were reported low with 2 and 1 in 10,000 patient years, respectively [6]. Children are in a more vulnerable position since they are at an increased risk for EBV seroconversion and will be exposed to therapies for a longer period of time. In the most current ECCO guidelines on prevention of infections in patients with IBD, EBV testing prior to immunosuppressive treatment, especially with thiopurines, and well considerations of thiopurine treatment in EBV-IgG negative patients, are suggested [25].

Another important aspect is the higher chance in pediatric CD patients to have unrecognized XIAP deficiency as in one patient of our cohort. With the onset of CD at the age of 8 years and an almost therapy-refractory course with severe perianal disease, it took two years until diagnostic testing for XIAP deficiency was initiated during an episode of HLH possibly triggered by EBV infection. Early functional and genetic testing for XIAP deficiency, especially in children who are difficult to treat with CD and VEO-IBD, might be an additional strategy to anticipate, and possibly prevent severe morbidity related to EBV infection [26,27].

In our cohort of 402 pediatric IBD patients, three patients developed a malignancy, but none seemed to be triggered by EBV. One patient, who received combination therapy of azathioprine and anti-TNFα, developed a Hodgkin lymphoma independent of EBV infection. It is known that patients treated with thiopurines are at a four-to-six-fold increased risk to develop a lymphoproliferative disorder, not all are related to EBV but associated with thiopurine treatment [3,4,5]. Another patient presented with a testicular germ cell tumor at the age of 18. He received anti-TNFα over the past six years and had, for the first time, shown a positive EBV-VCA-IgG serology six months prior to the diagnosis of malignancy. Recently, a significant association between EBV and testicular germ cell tumors was shown [28]. The third patient presented with colonic adenocarcinoma at the age of 19 years, while EBV serology was negative one year before.

Current ECCO and ESPGHAN guidelines for the management of UC and CD recommend thiopurines as therapeutic option for maintaining remission for CD and in moderate to severe UC, either as mono-therapy or as concomitant therapy to infliximab [29,30]. Especially in North America, there is a tendency to abandon thiopurines for the earlier use of TNFα inhibitors in consideration of the risks and benefits [31]. Overall, absolute cumulative risk for malignancies during therapy with thiopurines has been described as low [5], but treatment is associated with higher risk of EBV infection, as previously reported in children with IBD [12,32]. In a very recent study, Harris and colleagues also reported similar results on their cohort of children with IBD who had EBV infection. Independent of EBV status at the time of IBD diagnosis, a high proportion of children were treated with thiopurines (83.5%) and, during a mean follow-up time of 4.6 years, nine children with thiopurine therapy had proven EBV infection compared to three children without thiopurine treatment. In this cohort, in all of the primary EBV-infected children treatment with azathioprine was paused and subsequently restarted without any major complications reported, leading to the authors’ suggestion not to abandon thiopurine use as an effective maintenance treatment option for children with IBD [33]. We agree with Martinelli et al. [34] and Kucharzik et al. [25] and suggest that EBV serological testing should be performed prior to maintenance therapy and it should be carefully discussed with patients and their parents if thiopurine therapy should be used in their potentially EBV-naïve child, especially in male patients.

The strengths of this study are that we prospectively assessed the EBV seroconversion rates in a relatively large cohort with half of the children and adolescents being EBV-negative at the time of IBD diagnosis.

As a limitation, no serial follow-up testing is available, except in those who were tested for clinically suspected acute infection. Therefore, the exact age at the time of seroconversion remains unknown in children without clinical signs for mononucleosis. Since the overall number of patients in our study is relatively large, our cohort is too small to calculate the absolute risk ratios for EBV-associated comorbidities, such as HLH or malignancies. In addition, neither functional testing or genetic testing, especially for XIAP gene mutations [35], were broadly available in our cohort.

## 5. Conclusions

About 50% of pediatric IBD patients were EBV-naïve at diagnosis and 30% of pediatric IBD patients prior to their transfer to adult care were subject to increased risk to develop severe EBV infection during immunosuppressive therapy. Our data confirm the risk of developing HLH triggered by an acute EBV infection in children with IBD treated with thiopurines. This support the concept of testing for EBV status in children and young adults with IBD prior to starting maintenance therapy. If thiopurines are used in EBV n-ïve pediatric patients, parents and patients should be educated to contact the caring IBD physician in case of symptoms suggestive of mononucleosis to be investigated and closely monitored for signs of HLH. Functional or genetic testing for XIAP deficiency should be offered to male patients with EBV-related complications and those with therapy refractory severe CD-like disease.

## Figures and Tables

**Table 1:** Characteristics of patients with available EBV serology at IBD diagnosis (*n* = 194).

Factors	All Patients (*n* = 194)	EBV-Positive (*n* = 102)	EBV-Negative (*n* = 92)	*p*-Value
Males, *n* (%)	110 (56.7)	59 (57.8)	51 (55.4)	0.735 *
Age at IBD diagnosis				
Mean (SD)	11.3 (3.9)	12.0 (3.5)	10.4 (4.1)	
Median (IQR)	12.0 (9.0–14.0)	13.0 (10.0–15.0)	11.5 (8.0–14.0)	0.005 **
Age groups at IBD diagnosis, *n* (%) ^1^				0.040 *
0–9 years	56 (28.8)	22 (21.5)	34 (36.9)	
9–13 years	65 (33.5)	35 (34.3)	30 (32.6)	
13–18 years	73 (37.6)	45 (44.1)	28 (30.4)	
Diagnosis, *n* (%)				0.963 *
Ulcerative colitis	79 (40.7)	41 (40.2)	38 (41.3)	
Crohn’s disease	103 (53.0)	55 (53.9)	48 (52.1)	
IBD-Unclassified	12 (6.1)	6 (5.8)	6 (6.5)	

Table 1 shows characteristics of patients with available Epstein–Barr virus (EBV) serology at inflammatory bowel disease (IBD) diagnosis (*n* = 194). Results are represented in mean and standard deviation (SD) or median and interquartile range (IQR) ^1^ We considered the cut-offs for the age groups according to the 1. Quartile and median age in the cohort. * *p*-values refer to the comparison between the EBV-positive group and the EBV-negative group obtained by the Pearson’s Chi-square test. ** *p*-values obtained from the Wilcoxon range test.

**Table 2:** Univariate logistic regression model for factors associated with positive EBV serology in 194 patients with EBV status available at IBD diagnosis.

	OR *	(95% CI) *	*p*-Value **
Gender (Male vs. Female)	1.10	(0.63 to 1.95)	0.735
Age			
Age ≥ 13 vs. Age < 9 years	2.48	(1.21 to 5.07)	0.013
Age 9–13 vs. Age < 9 years	1.80	(0.87 to 3.72)	0.111
Diagnosis (Crohn’s disease vs. Ulcerative colitis)	1.06	(0.59 to 1.91)	0.841

Table 2 summarizes results from univariate logistic regression model for factors associated with Epstein–Barr virus (EBV) status at inflammatory bowel disease (IBD) diagnosis (*n* = 194). * Unadjusted odds ratios (OR) with a 95% confidence intervals (95% CI) obtained from the univariate logistic regression. ** *p*-values obtained from the Wald Chi-square test for the significance of OR.

**Table 3:** Univariate logistic regression model for factors associated with EBV seroconversion after IBD diagnosis (*n* = 66).

	OR *	(95% CI) *	*p*-Value **
Gender (Male vs. Female)	1.34	(0.37 to 4.91)	0.660
Age			
Age 13–16 vs. Age 3–13 years	0.32	(0.05 to 1.96)	0.217
Age 16–25 vs. Age 3–13 years	1.43	(0.32 to 6.39)	0.641
Diagnosis (Crohn’s disease vs. Ulcerative colitis)	1.15	(0.29 to 4.56)	0.842
Therapies			
5-aminosalicylic acid vs. no 5-aminosalicylic acid	1.78	(0.42 to 7.45)	0.431
Thiopurines vs. no thiopurines	3.71	(0.89 to 15.52)	0.073
Methotrexate vs. no methotrexate	0.22	(0.03 to 1.89)	0.169
Biologicals vs. no biologicals	0.49	(0.12 to 1.95)	0.311

Table 3 depicts results of Univariate logistic regression model for factors associated with Epstein–Barr virus (EBV) status at follow-up after diagnosis of inflammatory bowel disease (IBD) (*n* = 66). * Unadjusted odds ratios (OR) with 95% confidence intervals (95% CI) were obtained from the univariate logistic regression. ** *p*-values obtained from the Wald Chi-square test for the significance of OR.

## Data Availability

The data underlying this article will be shared on reasonable request to the corresponding author.

## Supplementary Materials

The following are available online at https://www.mdpi.com/article/10.3390/jcm10215187/s1, Table S1: Characteristics of all patients with at any time point available EBV serology (*N* = 207), Table S2: Characteristics of all patients with IBD in our cohort (*N* = 402). Table S3: Characteristics of patients with available follow up Epstein-Barr virus (EBV) serology (*N* = 66). Results are represented in mean and standard deviation (SD) or median and interquartile range (IQR). Figure S1: Flow chart of study population. Figure S2: Box plot for age at first inflammatory bowel disease (IBD) diagnosis by Epstein-Barr virus (EBV) status (*N* = 194), *p* = 0.005.
