# Supplementary material for: Epstein–Barr Virus Prevalence at Diagnosis and Seroconversion during Follow-Up in Pediatric Inflammatory Bowel Disease
Source: J Clin Med. 2021 Nov 6;10(21):5187. doi: 10.3390/jcm10215187 (PMC8584287; doi:10.3390/jcm10215187)

**Supplementary materials**

**Supplemental Table S1.** Characteristics of all patients with at any time point available Epstein-Barr virus (EBV) serology (N=207). Results are represented in mean and standard deviation (SD) or median and interquartile range (IQR).

**Supplemental Table 1** Characteristics of all patients with available EBV serology at any time point (N=207 of a total of 402)

| Factors | All patients (n=207) | EBV positive (n=110) | EBV negative (n=97) | P-value |
|---|---|---|---|---|
| **Males, n (%)** | 115 (55.6) | 64 (58.2) | 51 (52.6) | 0.418* |
| **Age at EBV testing** | | | | |
| Mean (SD) | 11.4 (4.0) | 12.2 (3.7) | 10.4 (4.2) | **0.001**** |
| Median (IQR) | 12.0 (9.0-14.0) | 13.0 (10.0-15.0) | 11.0 (8.0-14.0) | **0.006**** |
| **Age groups, n (%)** | | | | **0.018*** |
| 0-9 years | 61 (29.5) | 24 (21.8) | 37 (38.1) | |
| 9-13 years | 67 (32.4) | 36 (32.7) | 31 (32.0) | |
| 13-18 years | 79 (38.1) | 50 (45.5) | 29 (29.9) | |
| **Diagnosis, n (%)** | | | | 0.872* |
| Ulcerative colitis | 84 (40.6) | 45 (40.9) | 39 (40.2) | |
| Crohn's disease | 110 (53.1) | 59 (53.6) | 51 (52.6) | |
| IBD-unclassified | 13 (6.3) | 6 (5.5) | 7 (7.2) | |

*P-values refer to the comparison between EBV positive group and EBV negative group obtained by the Pearson's Chi-square test.
** P-values obtained from the Wilcoxon rank-sum test.

**Supplemental Table S2.** Characteristics of all patients with inflammatory bowel disease (IBD) in our cohort (N= 402). Results are represented in mean and standard deviation (SD) or median and interquartile range (IQR).

**Supplemental Table S2** Characteristics of all patients with IBD in our cohort (N= 402)

| **Factors** | **All patients (n=402)** | **EBV status available (n=207)** | **No EBV status available (n=195)** | **P-value** |
|---|---|---|---|---|
| **Males, n (%)** | 226 (56.2) | 115 (55.6) | 111 (56.9) | 0.782* |
| **Age at IBD diagnosis** | | | | |
| Mean (SD) | 11.0 (4.0) | 11.1 (4.0) | 11.0 (4.0) | **0.464**** |
| Median (IQR) | 12.0 (9.0-14.0) | 12.0 (9.0-14.0) | 12.0 (9.0-14.0) | **0.237**** |
| **Age groups, n (%)** | | | | **0.183*** |
| 0-9 years | 124 (30.9) | 65 (31.4) | 59 (30.3) | |
| 9-13 years | 148 (36.8) | 68 (32.9) | 80 (41.0) | |
| 13-18 years | 130 (32.3) | 74 (35.7) | 56 (28.7) | |
| **Diagnosis, n (%)** | | | | 0.275* |
| Ulcerative colitis | 153 (38.1) | 84 (40.6) | 69 (35.4) | |
| Crohn's disease | 228 (56.7) | 110 (53.1) | 118 (60.5) | |
| IBD-unclassified | 21 (5.2) | 13 (6.3) | 8 (4.1) | |

*P-values refer to the comparison between EBV positive group and EBV negative group obtained by the Pearson's Chi-square test.
** P-values obtained from the Wilcoxon rank-sum test.

**Supplemental Table S3.** Characteristics of patients with available follow up Epstein-Barr virus (EBV) serology (N=66). Results are represented in mean and standard deviation (SD) or median and interquartile range (IQR).

| **Supplemental Table 3** Characteristics of patients with available follow up EBV serology (N=66) | | | | |
|---|---|---|---|---|
| **Factors** | **All patients (N=66) No. (%)** | **EBV positive (n=11) No. (%)** | **EBV negative (n=55) No. (%)** | **P-value** |
| **Males, n (%)** | 32 (48.5) | 6 (54.5) | 26 (47.3) | 0.748 [b)] |
| **Age** | | | | |
| Mean (SD) | 14.5 (4.1) | 13.9 (4.5) | 14.7 (4.0) | 0.903 [c)] |
| Median (IQR) | 15.5 (13.0-17.0) | 16.0 (9.0-17.0) | 15.0 (13.0-17.0) | 0.743 [d)] |
| **Age groups** [a)] | | | | 0.223 [b)] |
| 0-13 years | 20 (30.3) | 4 (36.4) | 16 (29.1) | |
| 13-16 years | 27 (40.9) | 2 (18.2) | 25 (45.5) | |
| 16-25 years | 19 (28.8) | 5 (45.5) | 14 (25.5) | |
| **Follow up time Mean (SD)** | 4.3 (3.0) | 5.0 (4.2) | 4.2 (2.8) | 0.951 [c)] |
| **Diagnosis** | | | | 0.613 [b)] |
| Ulcerative colitis | 27 (40.9) | 4 (36.4) | 23 (41.8) | |
| Crohn's disease | 36 (54.5) | 6 (54.5) | 30 (54.5) | |
| IBD-unclassified | 3 (4.5) | 1 (9.1) | 2 (3.6) | |
| **Therapies** | | | | |
| Biologicals | 50 (75.8) | 7 (63.6) | 43 (78.2) | 0.440 [b)] |
| Thiopurines | 31 (47.0) | 8 (72.7) | 23 (41.8) | 0.097 [b)] |
| Methotrexate | 18 (27.3) | 1 (9.1) | 17 (30.9) | 0.265 [b)] |
| Combotherapy * | 24 (36.4) | 4 (36.4) | 20 (36.4) | 1.000 [b)] |
| 5-aminosalicylic acid | 41 (62.1) | 8 (72.7) | 33 (60.0) | 0.513 [b)] |

a) We considered the cut-offs according to the 1. Quartile and median age in the longitudinal cohort.

b) P-values refer to comparison between EBV positive and EBV negative group obtained by the Fisher’s Exact Test.

c) P-values obtained from Wilcoxon-Mann-Whitney test (Wilcoxon rank sum test).

d) P-values applied from Median test.

* Combination therapy of a biological and methotrexate or thiopurines.

**Supplemental Figure S1.** Flow chart of study population.

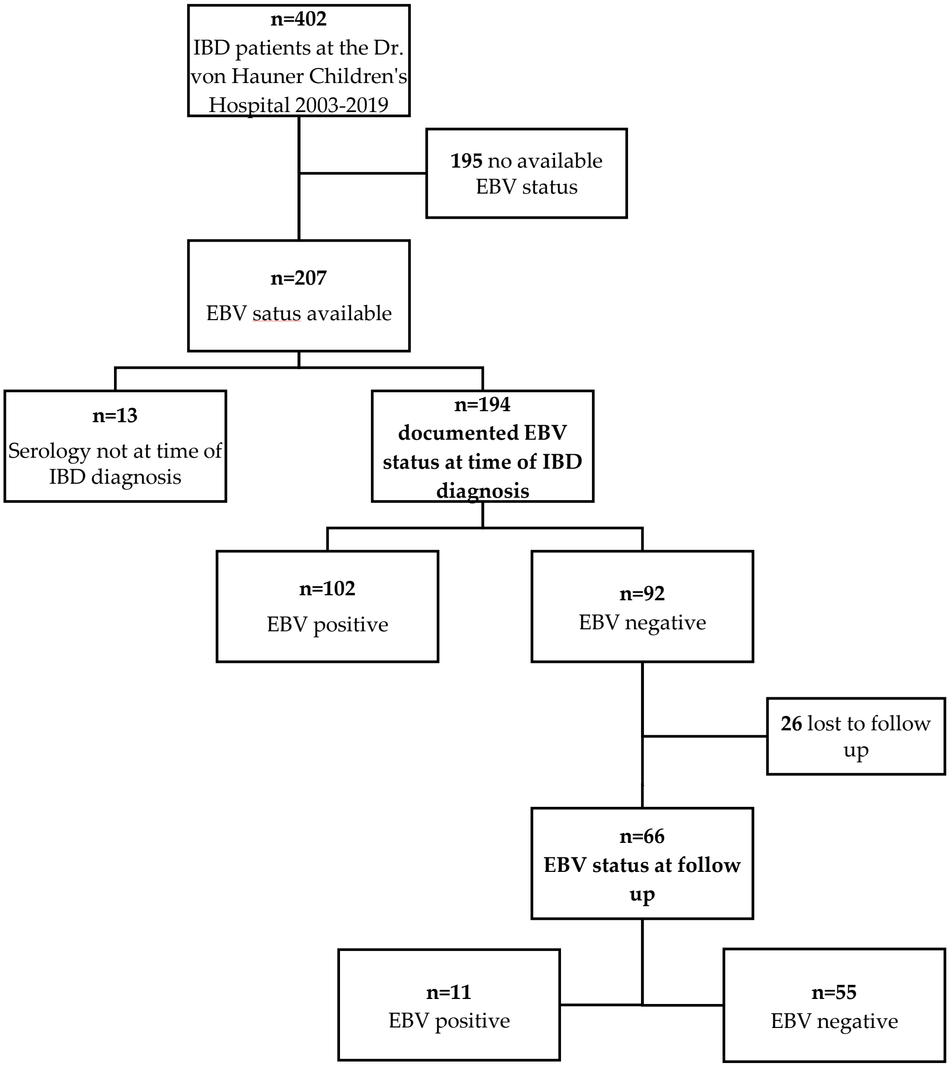

**Supplemental Figure S2.** Box plot for age at first inflammatory bowel disease (IBD) diagnosis by Epstein-Barr virus (EBV) status (N=194), p=0.005.

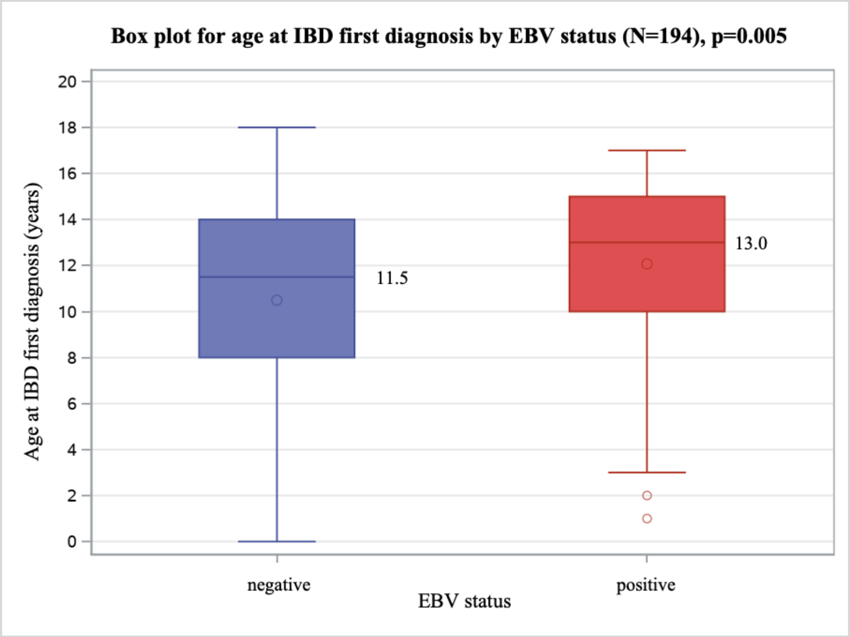

Supplement: Supplementary file 1 [file jcm-10-05187-s001.zip › jcm-1441027-supplementary.pdf]
